# Near-Infrared Spectroscopy for Monitoring Sternocleidomastoid Muscular Oxygenation during Isometric Flexion for Patients with Mild Nonspecific Neck Pain: A Pilot Study

**DOI:** 10.3390/s20082197

**Published:** 2020-04-13

**Authors:** Chia-Chi Yang, Po-Ching Yang, Jia-Jin J. Chen, Yi-Horng Lai, Chia-Han Hu, Yung Chang, Shihfan Jack Tu, Lan-Yuen Guo

**Affiliations:** 1 The Master Program of Long-Term Care in Aging, College of Nursing, Kaohsiung Medical University, Kaohsiung 807, Taiwan; chiachiyang@kmu.edu.tw; 2 Center for Long-Term Care Research, Kaohsiung Medical University, Kaohsiung 807, Taiwan; 3 Department of Sports Medicine, College of Medicine, Kaohsiung Medical University, Kaohsiung 807, Taiwan; s8784195@gmail.com (P.-C.Y.); jiaalhan@gmail.com (C.-H.H.); jackshtu@gmail.com (S.J.T.); 4 Department of BioMedical Engineering, College of Engineering, National Cheng Kung University, Tainan 701, Taiwan; chenjj@mail.ncku.edu.tw (J.-J.J.C.); changyung1205@hotmail.com (Y.C.); 5 Medical Device Innovation Center, National Cheng Kung University, Tainan 701, Taiwan; 6 School of Mechanical and Electrical Engineering, Xiamen University Tan Kah Kee College, Zhangzhou 361005, China; lai81.tom@gmail.com; 7 Ph. D. Program in Biomedical Engineering, College of Medicine, Kaohsiung Medical University, Kaohsiung 807, Taiwan; 8 Department of Medical Research, Kaohsiung Medical University Hospital, Kaohsiung 807, Taiwan

**Keywords:** nonspecific neck pain, muscular oxygenation, near-infrared spectroscopy, oxygenation oscillations

## Abstract

Since there is merit in noninvasive monitoring of muscular oxidative metabolism for near-infrared spectroscopy in a wide range of clinical scenarios, the present study attempted to evaluate the clinical usability for featuring the modulatory strategies of sternocleidomastoid muscular oxygenation using near-infrared spectroscopy in mild nonspecific neck pain patients. The muscular oxygenation variables of the dominant or affected sternocleidomastoid muscles of interest were extracted at 25% of the maximum voluntary isometric contraction from ten patients (5 males and 5 females, 23.6 ± 4.2 years) and asymptomatic individuals (6 males and 4 females, 24.0 ± 5.1 years) using near-infrared spectroscopy. Only a shorter half-deoxygenation time of oxygen saturation during a sternocleidomastoid isometric contraction was noted in patients compared to asymptomatic individuals (10.43 ± 1.79 s vs. 13.82 ± 1.42 s, *p* < 0.001). Even though the lack of statically significant differences in most of the muscular oxygenation variables failed to refine the definite pathogenic mechanisms underlying nonspecific neck pain, the findings of modulatory strategies of faster deoxygenation implied that near-infrared spectroscopy appears to have practical potential to provide relevant physiological information regarding muscular oxidative metabolism and constituted convincing preliminary evidences of the adaptive manipulations rather than pathological responses of oxidative metabolism capacity of sternocleidomastoid muscles in nonspecific neck patients with mild disability.

## 1. Introduction

Various epidemiological data indicates that neck pain has become a general reason that people seek medical assistance and a primary cause of disability worldwide [1,2,3,4,5,6]. Because of unequivocal anatomical structural abnormalities or identifiable orthopedic and neurological problems, it is commonly categorized as nonspecific neck pain [7,8]. Multifactorial origins, such as prolonged workloads, poor work postures and even psychological distress, are likely to be associated with this musculoskeletal condition [9,10,11]. Except for cervical kinematic and kinetic aberrations [12,13,14,15,16,17,18,19,20,21], previous electrophysiological findings have demonstrated the appearance of neuromuscular adaptations for patients with neck pain [22,23,24,25,26,27,28,29,30]. Increased activation of the superficial cervical flexor muscles and the upper trapezius [23,24,25,26,27,30] and reduced activation of the deep cervical flexor muscles [27,28,29] account for the compensatory modulation of hyper-activation of the superficial cervical flexor muscles for the weak or inhibited activation of the deep cervical flexor muscles in neck pain patients [25,26]. Also, patients with neck pain had neural recruitment strategies that showed relatively poor efficiency in the superficial flexor muscles when required motor tasks were executed [31,32], reflecting the phenomenon of greater fatigability of the superficial cervical flexor muscles [30,33].

Furthermore, near-infrared spectroscopy (NIRS) utilizes optical properties such as absorption and scattering to provide pathophysiological information from biological system. Briefly, near-infrared wavelength light between approximately 650 and 1000 nm is emitted into the muscle of interest from a source, and a detector tracks the attenuation of the intensity of the emitted light. Subsequently, both changes in the oxygenated and deoxygenated hemoglobin concentration could be further deduced from changes in the light intensity through algorithmic transformation governed by the modified Beer-Lambert law. Recent evidences have shown the potential of NIRS in various medical applications, including functional analysis of the brain and continuous monitoring of changes in local muscular oxygenation [34,35,36,37,38,39,40,41,42,43,44,45]. For example, functional NIRS has been used to monitor the time course of oxygenated/deoxygenated hemoglobin signal to estimate regional neural activity of the brain [36] and even could serve as an auxiliary diagnostic apparatus for distinguishing unipolar depression from schizophrenia and bipolar depression [42]. Similarly, the clinical applicability of NIRS for noninvasive evaluating of deconditioning and reconditioning of skeletal muscle oxidative functions has been emphasized [40], showing slower rates of reoxygenation of lower limb after exercise in patients with peripheral vessel disease [43], respiratory muscle hypoperfusion combined with the greater work of breathing in patients with congestive heart failure [44], functional restoration of muscle oxidative metabolism after renal transplantation [45] as well as regional heterogeneity in the distribution of blood flow and oxygen consumption in exercising muscle in patients with chronic obstructive pulmonary disease [38,39]. 

Because of the capability of NIRS for offering non-invasive monitoring of tissue oxygenation in a wide range of clinical scenarios, alternative potential modulatory mechanisms from a muscular oxidative metabolism viewpoint for neck-related neuromuscular conditions have also been considered [36,37,38,39,40,41,42,43,44,45,46,47,48,49]. Existing observation noted that muscular oxygen saturation levels in the trapezius descendens muscles were different between female workers with trapezius myalgia and healthy controls [49]. Another study investigated the effects of maximal isometric contractions on the oxygenation of the trapezius muscles in populations with chronic neck and shoulder pain and showed that trapezius muscles responding to isometric contractions were characterized by inferior oxygenation and blood flow [48]. However, a lack of studies that characterize the properties of muscular oxygenation for the neck flexor muscles in neck pain patients contributes to the lack of clarity regarding the pathogenic mechanisms underlying neck pain. Since there is merit in non-invasively monitoring muscular oxidative metabolism for NIRS in various clinical conditions [34,35,36,37,38,39,40,41,43,44,45], the present study mainly aimed at evaluating the clinical potential for characterizing the modulatory strategies of sternocleidomastoid muscular oxygenation by means of NIRS in patients with mild nonspecific neck pain. Further, we hypothesized either adaptive manipulations or pathological responses of oxidative metabolism capacity of sternocleidomastoid muscles in nonspecific neck patients with mild disability. Accordingly, we were particularly interested in examining whether sternocleidomastoid muscular oxygenation variables (including the oxygenated hemoglobin concentration and the deoxygenated hemoglobin concentration), the dynamic balance of oxygen delivery and consumption and the periodic nature of muscular oxygenation oscillations would be comparable between patients with mild nonspecific neck pain and asymptomatic individuals.

## 2. Materials and Methods

All the experimental procedures were approved by the Institutional Review Board of Kaohsiung Medical University Chung-Ho Memorial Hospital (No. KMUH-IRB-20120078) and performed in accordance with relevant guidelines of the Declaration of Helsinki. Prior to participation, all voluntary participants clearly understood the major objectives of the current and written informed consent was obtained from each individual participant. 

### 2.1. Subjects

Two groups of volunteers were enrolled: one group of ten participants with nonspecific neck pain (5 males, 5 females) who had sought medical treatment within the past 6 weeks with no medical histories of spinal neurological and orthopedic problems, such as spinal vertebral fracture, abnormal spinal lordosis, spinal spondylosis or spinal osteoarthritis. One group of ten participants (6 males, 4 females) with no history of cervical surgery, cervical trauma, cervical pain or neuromuscular problems. Anthropometric details were initially recorded. Next, the level of neck functional disability and adipose tissue thicknesses at the site of the probe were respectively assessed for each participant using the Neck Disability Index (NDI) [50] and skinfold caliper. The detailed demographic data of the participants are summarized in Table 1. Among these participants, patients with nonspecific neck pain, rated as having mild neck disability, had a higher NDI score than asymptomatic individuals (9.23 ± 4.00 vs. 2.08 ± 0.50, *p* < 0.01). Moreover, the groups did not differ in terms of age, gender distribution, weight, BMI and sternocleidomastoid skinfold thickness (*p* > 0.05).

### 2.2. Evaluation of Muscular Oxygenation Variables Using Near-Infrared Spectroscopy

In this investigation, the characteristics of sternocleidomastoid muscular oxygenation were continuously monitored during isometric flexion by a commercial frequency-domain NIRS system (Imagent, ISS Inc., Champaign, IL, USA) in real time at a 25 Hz sample rate. On the other hand, as previous work had mentioned that the frequency-domain multiple-distance system with over distances in the range of 1.5 to 4.5 cm could minimize the influence of between-subjects variations in adipose tissue thickness on NIRS signals and accurately quantify the optical properties of the superficial muscle [51], the multiple-channel NIRS probe was adopted to attenuate the confounding effects of adipose tissue thickness on NIRS measurements [52,53] and attached on the sternal head of the dominant or affected sternocleidomastoid muscle. The system had four sources and one detector with interoptode distances of 2.05, 2.55, 3.05 and 3.55 cm. Moreover, the adipose tissue thickness at the site of the probe was measured by a skinfold caliper (Beta Technology, Santa Cruz, CA, USA) and the measured adipose tissue thicknesses were 0.6 ± 0.2 mm for the nonspecific neck pain patients and 0.5 ± 0.2 mm for the asymptomatic individuals. The adipose tissue thickness was less than half of the source-detector separation [54] and the penetration depth of the NIRS signal therefore reasonably reflected the oxygenated and deoxygenated conditions of the testing muscle. In this work, emitted light at wavelengths of 690 and 850 nm was used to estimate the oxygenated hemoglobin concentration [HbO_2_], the deoxygenated hemoglobin concentration [Hb] and the total hemoglobin concentration [tHb] ([HbO_2_]+ [Hb] µM). 

**Figure 1:**
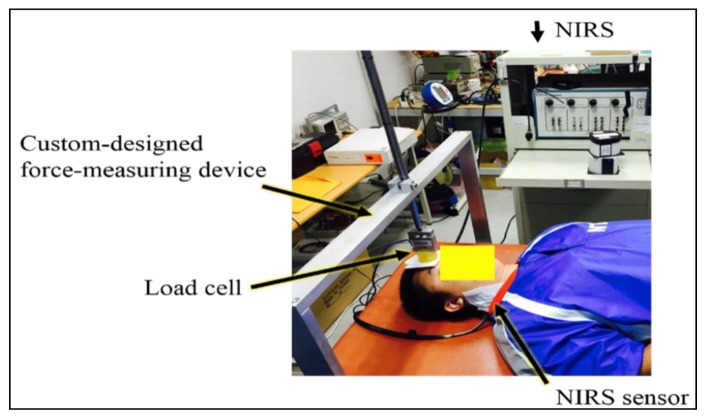
Experimental set-up for collecting muscular oxygenation variables of the dominant or affected sternocleidomastoid muscles by means of commercial NIRS system (Imagent, ISS Inc., Champaign, IL, USA) in real-time at a 25 Hz sample rate during isometric flexion.

After completing the experimental setup and instrument calibration to acquire the effective optical coefficients, all of the participants were familiarized with the experimental protocol followed by the conduction of three brief, voluntary, maximum isometric contractions for three seconds separated by a rest period of at least five minutes. The contraction with the highest exerted force recorded using custom-designed force-measurement device that consisted of a tension/compression minibean load cell was selected as the maximum voluntary contraction (MVC) and used for the calculation of the target levels for submaximal contractions. Next, all participants performed a trapezoid isometric muscle action of the sternocleidomastoid muscles, which displayed a linear increasing sternocleidomastoid isometric contraction from the baseline to 25% MVC at a rate of 10% MVC/s, a submaximal constant force of the target 25% MVC for 20 s, and a linear decrease back the baseline at 10%MVC/s. All recruited participants were instructed to maintain their force output as close as possible to the target force. Trials were repeated if the actual force production varied more than 5% from the target force and a sufficient rest period of at least ten minutes was provided between each trial to avoid muscle fatigue. A visual template and feedback trace of the force output during the trapezoid isometric muscle action and verbal encouragements were available to facilitate the achievement of the requested task (Figure 1).

### 2.3. Data Analysis of the Features of Sternocleidomastoid Muscular Oxygenation

All continuous muscular oxygenation variables including the oxygenated hemoglobin concentration [HbO_2_] and the deoxygenated hemoglobin concentration [Hb] were recorded. To further clarify the dynamic balance of O_2_ delivery and consumption of the target muscles during contraction and recovery periods, the StO_2_ was expressed using the following formula [51,52,55]:

(1) $${\mathrm{StO}}_{2}\left(\%\right)=\frac{\left[{\mathrm{HbO}}_{2}\right]}{\left[\mathrm{tHb}\right]}\times 100\%=\frac{\left[{\mathrm{HbO}}_{2}\right]}{\left[{\mathrm{HbO}}_{2}\right]+\left[\mathrm{Hb}\right]}\times 100\%\;$$

**Figure 2:**
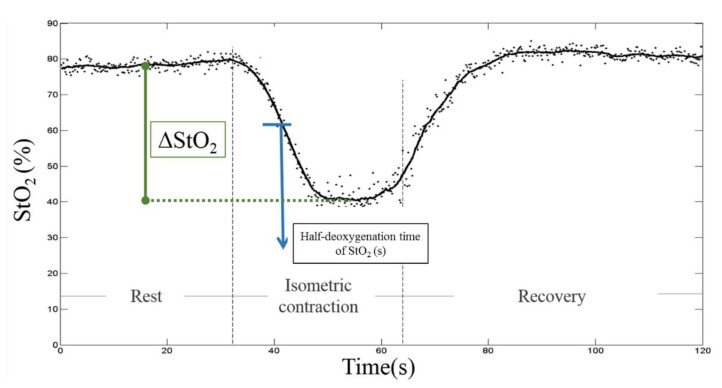
A representative example of time-domain analysis of muscular oxygenation kinetics. The oxygen saturation expressed as $\left(\left[{\mathrm{HbO}}_{2}\right]/\left[\mathrm{tHb}\right]\right)\times 100\%$ was adopted and a hyperbolic tangent equation with the least-square error method was applied to fit the oxygen saturation curves as a function of time during the contraction and recovery periods.

A hyperbolic tangent equation (tanh) was applied to fit the oxygen saturation curves (Y) as a function of time (t) during the contraction and recovery periods to constitute muscular oxygenation kinetics [43] (Figure 2):

(2) Y=a ×tanh(b×t−c)+d 

Using the least-square error method, the best-fit coefficients, a, b c and d of the muscular oxygenation kinetics were extracted. ΔStO_2_ (%) was estimated from the maximum and minimum oxygen saturation values of while the requested task was performed from the parameters d - a and d + a, respectively. From the two derived coefficients, c/b, which represents half-deoxygenation time of StO_2_, implying the flection time taken to reach 50% of the maximal oxygen consumption during the contraction phase, while it stands for half-reoxygenation time of StO_2_ in the recovery phase, accounting for the time interval from the end of a contraction to the time that the muscular oxygenation returns to the half-baseline value [55].

In addition to the time-domain view, the periodic nature of muscular oxygenation oscillations during the contraction and recovery period was further observed using a fast Fourier transform (FFT) algorithm. Before the FFT analysis, raw StO_2_ data were multiplied with a Hanning window and padded with zeros to 7500 data points to increase the resolution of the frequency spectrum. Next, the features of spontaneous oscillations of muscular oxygenation, such as the peak and median frequencies, were extracted to clarify the potential modulatory mechanisms of sternocleidomastoid muscular oxygenation in nonspecific neck pain condition. All of data analysis was processed using self-developed computational code in the M MATLAB programming language (R2015^®^, the Mathworks, Inc., Natick, MA, USA).

### 2.4. Statistical Analysis

Statistical procedures were performed with commercial Statistical Package for the Social Science software (SPSS 20.0^®^, IBM Corporation, Armonk, NY, USA). Descriptive statistics were used to characterize the group means and standard deviations for the demographic data and muscular oxygenation variables of interest for participants with nonspecific neck pain and asymptomatic individuals. Because of non-normal distribution of these variables verified by the Kolmogorov–Smirnov test, a nonparametric Mann–Whitney *U* test was chosen to further determine whether differences existed between the two tested populations. The results were considered to be statistically significant if the *p*-value was below 0.05.

## 3. Results

### 3.1. Muscular Oxygenation Variables of Interest

**Figure 3:**
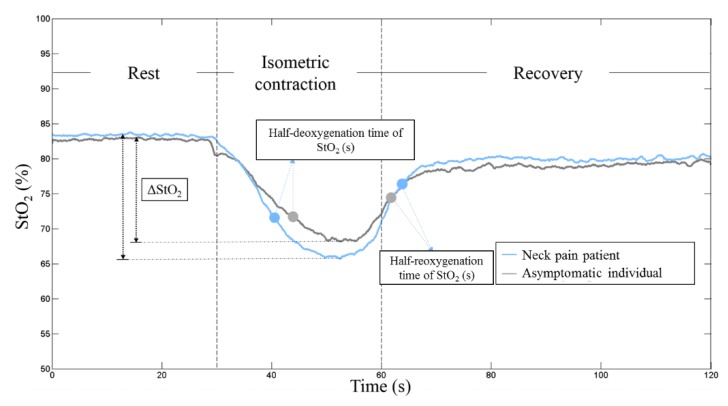
Representative time course of the oxygen saturation kinetics for the sternocleidomastoid muscle from one patient with nonspecific neck pain and an asymptomatic individual. ΔStO_2_ (%) was estimated from the maximum and minimum values of oxygen saturation while performing the requested task.

A representative time course of the oxygen saturation kinetics for the sternocleidomastoid muscle from a patient with nonspecific neck pain and an asymptomatic individual is shown in Figure 3. Table 2 summarizes the muscular oxygenation variables of interest for both groups. Only the half-deoxygenation time of oxygen saturation (StO_2_), which means the flection time taken to reach 50% of the maximal oxygen consumption during the contraction phase [53] in patients with nonspecific neck pain is shorter than asymptomatic individuals (10.43 ± 1.79 s vs. 13.82 ± 1.42 s, *p* < 0.001), but no any statistically significant differences in other muscular oxygenation variables (*p* > 0.05) were observed between these two groups. On the other hand, there were also no any differences between males (pooled data of neck pain and asymptomatic individuals) and females (pooled data of neck pain and asymptomatic individuals) in adipose tissue thickness, and muscular oxygenation variables (Table S1 of Supplementary Material).

### 3.2. Frequency Spectrum of Muscular Oxygenation Oscillations

**Figure 4:**
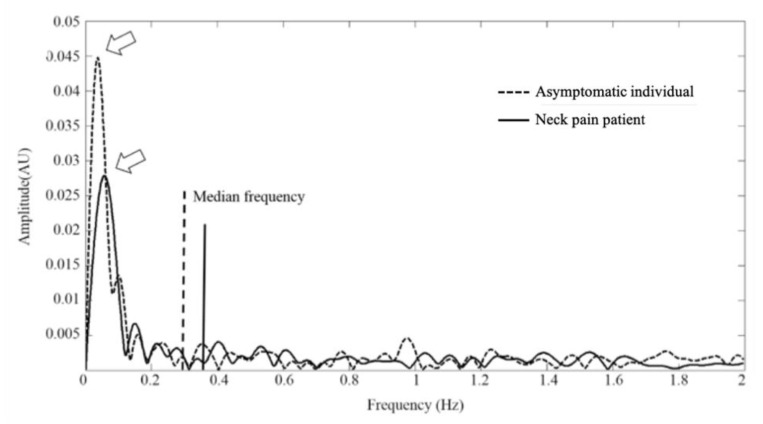
Comparison of the frequency spectrum of muscular oxygenation oscillations from one patient with nonspecific neck pain and asymptomatic individual. Arrowheads and vertical lines represent the lower-frequency distinctive peak at less 0.1 Hz and median frequency of muscle oxygenation oscillation from one patient with nonspecific neck pain and asymptomatic individual, respectively.

Except for time-domain analysis of oxygen saturation kinetics, the periodic nature of muscular oxygenation oscillations was further investigated using spectral analysis. Figure 4 is illustrative comparison of the frequency spectrum of muscular oxygenation oscillations from a patient with nonspecific neck pain and an asymptomatic individual. The pattern of muscular oxygenation oscillations between the nonspecific neck pain and asymptomatic groups was analogous and distinctive peak with a low-frequency of less 0.1 Hz could be observed. Participants with nonspecific neck pain had the detectable higher median frequency of muscular oxygenation oscillations (0.35 ± 0.10 vs. 0.29 ± 0.17), but the difference did not reach statistical significance (*p* = 0.436).

## 4. Discussion

The present study attempted to evaluate the clinical usability for featuring the modulatory strategies of sternocleidomastoid muscular oxygenation in nonspecific neck pain patients with mild neck disability. We, therefore, testified whether sternocleidomastoid muscular oxygenation variables, the dynamic balance of oxygen delivery and consumption and the periodic nature of muscular oxygenation oscillations would be comparable between patients with mild nonspecific neck pain and asymptomatic individuals. Even though the lack of statically significant differences in most of the muscular oxygenation variables, including baseline StO_2_, oxygen extraction, half-reoxygenation time of StO_2_ and patterns of the spontaneous oscillations of muscular oxygenation between nonspecific neck pain patients and asymptomatic individuals failed to refine the definite pathogenic mechanisms, the findings of the shorter half-deoxygenation time of StO_2_ implied that NIRS appears to have practical potential to provide relevant physiological information regarding muscular oxidative metabolism for mild nonspecific neck pain patients. It could be deduced from our findings that the mild pathological condition for the recruited nonspecific neck patients was not associated with oxidative metabolism capacity of sternocleidomastoid muscles. Most importantly, neck pain patients with mild disability displaying shorter half-deoxygenation time of StO_2_ and detectable but not statistically significant increase in the median frequency of muscular oxygenation oscillations soundly constituted preliminary evidences for the appearance of adaptive strategies of muscular oxygenation while executing a sternocleidomastoid isometric contraction.

On the top of that, the nonspecific neck pain patients displayed shorter half-deoxygenation time of StO_2_ of the sternocleidomastoid muscle in contrast to asymptomatic individuals, reflecting a need for greater oxygen extraction. Now that another important finding from the present work revealed comparable changes in oxygen saturation between the nonspecific neck pain and asymptomatic groups, it is reasonably inferred that the nonspecific neck pain patients would be likely to raise the capacity of muscle perfusion to achieve the acceleration of oxygen delivery, again hinting an adaptive mechanism to sustain a balance between oxygen delivery and utilization in sternocleidomastoid muscle.

Previous efforts indicated the physiological relevance of muscular oxygen metabolism for muscle function in healthy and pathological conditions, and it is generally thought that adaptive manipulations of muscular oxygenation are associated with various neuromuscular deficiencies [35,48,49,56,57,58,59,60,61]. Reduced muscular oxygenation and longer half-reoxygenation time of StO_2_ of the erector spinae muscle have been demonstrated in patients with low back pain [60,61]. Likewise, several reports also claimed larger decreases in the oxygenation of the trapezius muscles during repetitive pegboard tasks in female workers with trapezius myalgia [49] and indicated a lower oxygenation and a prolonged half-reoxygenation time of StO_2_ of the trapezius muscles in neck pain patients after one set of isometric exercises was executed [48,56]. These clinical findings implied that the affected muscles around an injured area would be more likely have an inferior capacity to consume and utilize oxygen in neuromuscular deficiencies. Unexpectedly, the current observation indicated that no definite evidence could account for the significant differences in oxygen extraction during contraction and the half-reoxygenation time of StO_2_ after contraction of the sternocleidomastoid muscles between the nonspecific neck pain and asymptomatic groups. Different muscles and muscular pathogenic conditions investigated in both the current and previous studies would interpret these discrepancies. Additionally, the enrollment of experimental volunteers with mild neck disability in the present work was likely to result in rather unexpected results. 

Although our findings were contrary to common hypotheses of disturbed muscular oxygenation characteristics associated with neuromuscular deficiencies [35,48,49,56,57,58,59,60,61], the present results somewhat tallied with the discoveries regarding the response of muscular oxygenation in work-related muscle pain [47]. In previous investigation, patients with work related muscle pain had a poorer muscular endurance ability and became fatigued earlier than the healthy controls, but no apparent group differences in the responses of the StO_2_ of the extensor carpi radialis and the trapezius muscles during low level sustained muscular contractions were detected. The findings of no group differences in oxygen extraction during contraction found from the current studies complied with the proven inference, which stated that early pathogenic condition or fatigue of patients with work-related muscle pain did not seem to be associated with muscular oxygenation and hemodynamics [47].

A noteworthy issue for discussion is the relevant role of oxygen availability for modulating motor unit recruitment and discharge patterns of activated motor units during contraction [62,63]. It is generally accepted that regardless of newly recruited motor units or an increased firing rate of already activated units, the rate of muscular oxygen utilization and consumption would increase as the increased excitatory drive of the motor units of a working muscle [56]. Since altered neuromuscular control strategies involving elevated initial firing rates of activated motor units for withstanding muscle tone in neck pain patients had proven [31], in conjunction with the current findings of shorter half-deoxygenation time of StO_2_ while initiating contraction, a conceivable modulatory strategy of sternocleidomastoid muscular oxygenation in nonspecific neck pain patients with mild neck disability could be deduced. The adaptive manipulation of faster oxygen delivery may be due to urgent requirement of oxygen supply for facilitating the augmentation of the initial discharge rates of the activated motor units. 

Further, the periodic nature of muscular oxygenation oscillations was characterized and approximately analogous patterns of the spontaneous oscillations of muscular oxygenation between the nonspecific neck pain and asymptomatic groups were identified. Specifically, the appearance of a low-frequency distinctive peak at less 0.1 Hz was noted in accordance with previous finding [57,64], which implied that the blood flow of the testing muscle was not severely occluded [65]. Furthermore, a nearly parallel trend of the median frequencies of muscular oxygenation oscillations was present in the nonspecific neck pain and asymptomatic groups. Past researches had shown effects of various physiological conditions, such as endothelial related metabolic, neurogenic and intrinsic myogenic activities on spontaneous oscillatory manifestations of muscular oxygenation [66,67,68] The amplitude of muscular oxygenation oscillations could mirror the activity level of these physiological origins [68] and the more significant low-frequency muscular oxygenation oscillations represented a higher activity of physiological origins [55]. Reduced activity of physiological origins, such as neurogenic or myogenic activity resulting from aging effects would also disturb the regulations of muscular oxygenation oscillations [68]. In other words, despite lack of statically significant differences in most of the features of muscular oxygenation oscillations between nonspecific neck pain patients and asymptomatic individuals herein, the detectable increase in the median frequency of muscular oxygenation oscillations (0.35 ± 0.10 for nonspecific neck pain patients vs. 0.29 ± 0.17 for asymptomatic individuals), to a certain extent, would also infer the occurrence of an adaptive manipulation of physiological origins in mild pathogenic condition of nonspecific neck pain during a sternocleidomastoid isometric contraction. 

Since the proposed frequency-domain NIRS system possesses practical potential for featuring the characteristics of the modulatory strategies of sternocleidomastoid muscular oxidative metabolism in mild nonspecific neck pain, comparing the characteristics of sternocleidomastoid muscular oxidative metabolism between nonspecific neck pain patients and asymptomatic individuals provide an auxiliary approach to differentiate on earth adaptive manipulations or pathological responses of oxidative metabolism capacity of sternocleidomastoid muscles in nonspecific neck patients with mild disability. Even the understanding of the characteristics of sternocleidomastoid muscular oxidative metabolism in nonspecific neck pain with different severities allow guiding the clinicians to evaluate the extent of impairment of the cervical spine and monitor the efficacy of rehabilitation programs in clinical practice.

Besides, because of the limited sample size and the enrollment of patients with mild neck disability, the significance relating to the preliminary findings from the current work and the comparisons thereof is exploratory and not confirmative. Next, using muscle contraction force instead of direct electromyography assessment for ensuing muscle activation level is another methodological limitation. For these reasons, the present findings should be interpreted with more caution. Further research is warranted to resolve these deficiencies and comprehensively elucidate the pathogenic mechanisms of neck pain.

## 5. Conclusions

Taken together, except for cervical kinematic and kinetic assessments, potential pathological mechanisms from a muscular oxidative metabolism viewpoint for neck-related neuromuscular conditions could be considered alternatively since the preliminary proof of clinical applicability of NIRS for noninvasive monitoring local muscular oxygenation. Another key point to mention is that, even if nearly sound oxidative metabolism capacity of sternocleidomastoid muscles, as evidenced by no statistically significant differences in most of the features of muscular oxygenation variables between the neck pain and asymptomatic groups, the current work corroborated a shorter half-deoxygenation time of StO_2_ during a sternocleidomastoid isometric contraction in the nonspecific neck pain patients. It could be at least partly speculated the adaptive manipulations rather than pathological responses of oxidative metabolism capacity of sternocleidomastoid muscles in nonspecific neck patients with mild disability. In addition, in keeping with our previous finding of the reinforcement of motor unit recruitment firing rates [31], it was inferred that, in order to achieve the prescribed tasks, the adaptive modulation of faster oxygen delivery would likely be responsible for rapidly supplying sufficient oxygen to the target muscle, which, in turn, would facilitate the augmentation of the initial discharge rates of the activated motor units for initiating the contraction of the stiffer sternocleidomastoid muscles in the mild pathogenic condition of nonspecific neck pain.

## Figures and Tables

**Table 1:** Characteristics of the demographic data for patients with nonspecific neck pain and asymptomatic individuals.

	Neck Pain Patients	Asymptomatic Individuals	*p*-Values
**Age (years)**	23.6 ± 4.2	24.0 ± 5.1	0.756
**Gender**	5 males, 5 females	6 males, 4 females	0.581
**Height (m)**	168.5 ± 7.5	165.1 ± 8.0	0.302
**Weight (kg)**	65.5 ± 15.8	61.5 ± 11.3	0.550
**BMI (m^2^/kg)**	22.9 ± 4.9	22.5 ± 3.7	0.943
**Skinfold thickness (mm)**	0.6 ± 0.2	0.5 ± 0.2	0.334
**NDI score**	9.2 ± 4.0	2.1 ± 0.5	0.008

**Table 2:** Muscular oxygenation variables of interest for nonspecific neck pain and asymptomatic groups.

	Neck Pain Patients	Asymptomatic Individuals	*p*-Values
**Baseline StO_2_ (%)**	83.57 ± 2.81	84.97 ± 3.22	0.353
**ΔStO_2_ (%)**	16.18 ± 5.58	15.41 ± 7.61	0.912
**Half-deoxygenation time of StO_2_ (s)**	10.43 ± 1.79	13.82 ± 1.42	<0.001
**Half-reoxygenation time of StO_2_ (s)**	7.66 ± 2.96	6.20 ± 2.50	0.393
**Median frequency (Hz)**	0.35 ± 0.10	0.29 ± 0.17	0.436

## Supplementary Materials

The following are available online at https://www.mdpi.com/1424-8220/20/8/2197/s1, Table S1: Adipose tissue thickness and muscular oxygenation variables of interest for male and female individual.
