# Supplementary material for: Near-Infrared Spectroscopy for Monitoring Sternocleidomastoid Muscular Oxygenation during Isometric Flexion for Patients with Mild Nonspecific Neck Pain: A Pilot Study
Source: Sensors (Basel). 2020 Apr 13;20(8):2197. doi: 10.3390/s20082197 (PMC7218888; doi:10.3390/s20082197)
Supplement: Supplementary file 1 [file sensors-20-02197-s001.pdf]

*sensors*

Supporting Information

# Near-Infrared Spectroscopy for Monitoring Sternocleidomastoid Muscular Oxygenation during Isometric Flexion for Patients with Mild Nonspecific Neck Pain: A Pilot Study

**Chia-Chi Yang [1,2], Po-Ching Yang [3], Jia-Jin J. Chen [4,5], Yi-Horng Lai [6], Chia-Han Hu [3], Yung Chang [4], Shihfan Jack Tu [3] and Lan-Yuen Guo [2,3,7,8,*]**

[1] The Master Program of Long-Term Care in Aging, College of Nursing, Kaohsiung Medical University, Kaohsiung, Taiwan; chiachiyang@kmu.edu.tw
[2] Center for Long-Term Care Research, Kaohsiung Medical University, Kaohsiung 807, Taiwan
[3] Department of Sports Medicine, College of Medicine, Kaohsiung Medical University, Kaohsiung 807, Taiwan; s8784195@gmail.com (P.-C.Y.); jiaalhan@gmail.com (C.-H.H.); jackshtu@gmail.com (S.J.T.)
[4] Department of BioMedical Engineering, College of Engineering, National Cheng Kung University, Tainan 701, Taiwan; chenjj@mail.ncku.edu.tw (J.-J.J.C.); changyung1205@hotmail.com (Y.C.)
[5] Medical Device Innovation Center, National Cheng Kung University, Tainan 701, Taiwan
[6] School of Mechanical and Electrical Engineering, Xiamen University Tan Kah Kee College, Fujian 361005, China; lai81.tom@gmail.com
[7] Ph. D. Program in Biomedical Engineering, College of Medicine, Kaohsiung Medical University, Kaohsiung 807, Taiwan
[8] Department of Medical Research, Kaohsiung Medical University Hospital, Kaohsiung 807, Taiwan
* Correspondence: yuen@kmu.edu.tw; Tel.: +886-7-3121101-2646-614

Received: 28 February 2020; Accepted: 8 April 2020; Published: 13 April 2020

Table S1. Adipose tissue thickness and muscular oxygenation variables of interest for male and female individual

| | **Male** (pooled data of neck pain and asymptomatic individuals) | **Female** (pooled data of neck pain and asymptomatic individuals) | ***P*-values** |
|---|---|---|---|
| **Adipose tissue thickness (mm)** | 0.54 ± 0.19 | 0.56 ± 0.25 | 0.882 |
| **Baseline $StO_2$ (%)** | 83.30 ± 3.41 | 84.97 ± 3.22 | 0.882 |
| **$\Delta StO_2$ (%)** | 14.67 ± 7.10 | 17.17 ± 5.80 | 0.456 |
| **Half-deoxygenation time of $StO_2$ (s)** | 12.52 ± 2.32 | 11.63 ± 2.42 | 0.456 |
| **Half-reoxygenation time of $StO_2$ (s)** | 6.14 ± 2.68 | 7.91 ± 2.34 | 0.230 |
| **Median frequency (Hz)** | 0.33 ± 0.14 | 0.32 ± 0.14 | 0.710 |

Data are shown as mean ± standard deviation. Because of non-normal distribution of these variables verified by the Kolmogorov-Smirnov test, a non-parametric Mann-Whitney *U* test was chosen to further determine whether differences existed between the two tested populations. The results were considered to be statistically significant if the *P*-value was below 0.05.

© 2020 by the authors. Licensee MDPI, Basel, Switzerland. This article is an open access article distributed under the terms and conditions of the Creative Commons Attribution (CC BY) license (http://creativecommons.org/licenses/by/4.0/).
